# Genetic and Familial Environmental Effects on Suicide – An Adoption Study of Siblings

**DOI:** 10.1371/journal.pone.0077973

**Published:** 2013-10-17

**Authors:** Liselotte Petersen, Thorkild I. A. Sørensen, Per Kragh Andersen, Preben Bo Mortensen, Keith Hawton

**Affiliations:** 1 National Centre for Register-based Research, University of Aarhus, Aarhus, Denmark; 2 National Centre for Register-based Research, University of Aarhus, Aarhus V, Denmark; 3 Novo Nordisk Foundation Center for Basic Metabolic Research, Faculty of Health and Medical Sciences, University of Copenhagen, Copenhagen, Denmark; 4 Department of Biostatistics, University of Copenhagen, Copenhagen, Denmark; 5 Centre for Suicide Research, University Department of Psychiatry, University of Oxford, Warneford Hospital, Oxford, United Kingdom; University of Iowa Hospitals & Clinics, United States of America

## Abstract

**Background:**

While there is clear evidence of familial influences on suicide, the origin of these is less certain. We have investigated genetic and familial environmental factors by studying the occurrence of suicide in biological and adoptive siblings of adoptees who died by suicide compared to siblings of surviving adoptees.

**Method:**

We used the Danish Adoption Register and Danish population registers to compare 221 siblings of adoptees who died by suicide with the siblings of 1,903 adoptees who did not die by suicide. All adoptions in the Danish Adoption Register are non-familial, i.e. the adoptive parents are biologically unrelated to the adoptee. Analyses were conducted on incidence rates of suicide in biological and adoptive siblings given occurrence of suicide in the adoptees while also taking into account psychiatric disorders.

**Results:**

The risk of suicide in full siblings of adoptees who died by suicide before age 60 years was significantly higher than in full siblings of adoptees who had not died by suicide (incidence rate ratios (IRR) = 5.01; 95% confidence interval [CI] = 1.28 - 19.6). This increase persisted after adjustment for history of psychiatric admission of siblings (IRR = 4.19; 95% CI = 1.00 - 17.5).

**Conclusions:**

Genetic factors influence risk of suicide, probably independently of psychiatric disorder. This is relevant in provision of advice to families, including possible prevention of suicide.

## Introduction

Studies of potential genetic effects on risk for suicide are based on investigations of twins and of adoptees and their families. Twin studies have shown that concordance for suicide is significantly more frequent in monozygotic than in dizygotic twins [1-3], suggesting genetic contributions to suicide. However, drawing this conclusion from twin studies assumes that the common environmental influences *in utero* and due to living in the same families, as well as the total genetic variance are identical in mono- and dizygotic pairs [4]. Moreover, genetic influences may be additive (i.e. due to different genetic variants) typically being expressed in parents and their offspring or non-additive (i.e. dominance effects by genetic variants or by interactions among genes), expressed among full siblings [4]. An example of a non-additive effect was found in an earlier adoptions study; analysing incidence and case-fatality of infectious disease in adoptees and their siblings there appeared to be a strong non-additive genetic influence on risk of fatal outcome [5]. In twin studies common environmental influences cannot be distinguished from non-additive genetic influences [4]. Unfortunately, there are extremely limited data on suicide risk in twins reared apart compared to those reared together, which might provide information on the contribution of both common environmental effects and genetic effects [3]. 

A large Swedish study investigated familial clustering of suicide risk in, among others, full- and half-siblings, parents and their offspring and also in spouses and adopted children [6]. This confirmed that there is a genetic influence contributing to the familial clustering of suicide risk. Further, based on higher risk among full siblings compared to parents and their offspring pairs and higher risk in maternal half siblings than in paternal half-siblings, they concluded that shared environment contributes to the familial risk of suicide. However, a stronger correlation between full siblings than between parents and their offspring could be due to either shared environment or to non-additive genetic effects [4]. Also, the stronger correlation in maternal half-siblings than in paternal half-sibling could be caused by either shared environment before as well after birth or it could be due to non-paternity for a proportion of those registered as paternal half-siblings. These alternative explanations could neither be proved nor disproved in the adopted children in the Swedish study.

Investigation of adoptees and their biological and adoptive parents and siblings provide powerful means of investigating genetic and familial environmental influences on risk of diseases. This has shown, for example, that premature death in adults, especially due to infection and vascular causes, has a strong genetic background [7,8]. We chose this approach for the present study. 

An earlier adoption study of suicide, based on the Danish Adoption Register, demonstrated that suicide risk was significantly increased in the biological parents and siblings of adoptees who died by suicide compared to the biological parents and siblings of adoptees who did not die by suicide [9]. This study was, however, limited to adoptees from the Copenhagen area (with just 57 adoptee suicides). Moreover, it did not distinguish between parents and siblings of the adoptees, and the analysis did not take into account clustering in families. The Swedish Multi-Generational register has been used to identify large groups of adoptees, born between 1946 and 1986, and their parents. This showed a two-fold increased risk of suicide in adoptees whose biological parents had died by suicide [10]. When adopted by relatives in the biologically-related family, the adoptee is brought up in a family environment that is not clearly separated from their genetic background. Investigations of non-familial adoptions, as in the Danish Adoption Register, enable better separation of genetic and familial environmental effect. Restricting analyses to siblings would also enable better assessment of non-additive genetic effects and reduce the potentially confounding influences from generational changes. 

Severe psychiatric disorders necessitating psychiatric hospital admission are strongly associated with risk of suicide [11-13]. Schulsinger and colleagues found evidence of genetic influence among people dying by suicide who did not have a history of psychiatric hospitalization as well as among those with such a history [9]. They discussed whether this suggests a pathogenic factor contributing to suicide independent of psychiatric disease, or whether suicide reflects an acute and severe psychiatric illness manifesting as suicide. A large Danish study strongly suggested that familial history of psychiatric illness increases suicide risk only through increasing the risk of developing a mental disorder. It also showed that family suicide history significantly increases suicide risk independently of familial history of psychiatric illness [14].

Using the national Danish Adoption Register and Danish Health Registers, we have estimated risk of suicide in biological full siblings and half-siblings and in adoptive siblings of adoptees. We compared the risk in siblings of adoptees who had died by suicide with the risk in siblings of surviving adoptees. The analyses include examination of risk according to history of psychiatric treatment. Our hypothesis was that our findings for suicide would provide evidence that genetically-transmitted risk (i.e. observed in biologically-related siblings) and familial environmentally-determined risk (i.e. observed in adoptive siblings) are present, and that these would occur irrespective of whether or not there was a history of psychiatric treatment.

## Study Population and Methods

### Adoption cohort and study design

The study was based on the Danish Adoption Register, which contains records on all 14,425 non-familial adoptions formally granted in Denmark during the period 1924 through 1947 [15]. We included adoptees born in 1917 or later who were traceable, were transferred to the adoptive family before the age of 7 years (25% were transferred directly after birth and mean age at transfer were 8 months), were singletons (or one from each twin pair), and who did not emigrate or die before the age of 12 years. There were 13,061 such adoptees, of whom 221 died by suicide before 31 December 2006. Except for persons not fulfilling our inclusion criteria (e.g. born before 1917 and transfer after age 7 years), our study included cases from the earlier adoption study by Schulsinger et al. [9].

From the Adoption Register, we also selected a sub-cohort consisting of a random sample of 1,933 adoptees, originally designed as an equally sized control sample in a study of the general mortality in this cohort [16]. We excluded the 30 suicides and used the others to represent adoptees who had not died by suicide. Adoptive and biological siblings were traced in the regionally organized civil registers, using information about their parents. Adoptive siblings are children growing up in the same family but not biologically related to the adoptee. Biological full siblings have the same mother and father as the adoptee, and half siblings have the same mother or father as the adoptee, but these biological siblings grew up either with their parents or in another adoptive family. Twins would be adopted together, so they were left out of the sampled siblings, whereas full siblings and half siblings adopted away were placed in separate adoptive families in 99.8 % of the cases. This means that the biological siblings in our study did not grow up together with the adoptee.

### Assessment of suicide, and psychiatric admissions

The adoptees and their biological and adoptive siblings were followed until death or 31 December 2009. Suicide was determined as the mode of death when the cause of death was coded as 900-940 (ICD 4-5) for the period 1942-49, as 970-980 (ICD 6-7) for the period 1950-68, as E950-959 (ICD-8) for the period 1969-93, and as X60-84 (ICD-10) from 1994 and onwards. For deaths occurring prior to 1942 the individual death certificates were examined and the causes classified by the investigators independent of specific classification systems. Information on psychiatric admissions defined as discharges, available from 1970 until 1 April 2010, was obtained by record linkage.

### Comparing of two cohorts

The rates of suicide in two cohorts of siblings were compared; one cohort included siblings of adoptees dying by suicide, the other cohort included siblings of adoptees not dying by suicide. When comparing the two cohorts, the interpretation differs between biological and adoptive siblings: adoptive siblings are biologically unrelated but grow up with the adoptee. Any association would indicate environmental influence. Biological siblings are genetically related but grew up either with their parents or in another adoptive family, not together with the adoptee. An association indicates genetic influence. In the primary analyses, the first cohort included siblings of adoptees dying by suicide before the age of 60 years, and the comparison cohort included siblings of adoptees known to survive to the age of 60 years (thus siblings of adoptees who died from other causes or emigrated before that age could not be included in either cohort). The reason for choosing a cut-off at age 60 years was that all surviving adoptees reached the age of 60 years by 2006, i.e. then their siblings could be included in the comparison cohort. It was possible to trace adoptive and/or biological siblings in the nationwide regionally organized civil registers for 173 adoptees dying by suicide and 1403 alive at age 60 years. 

**Figure 1:**
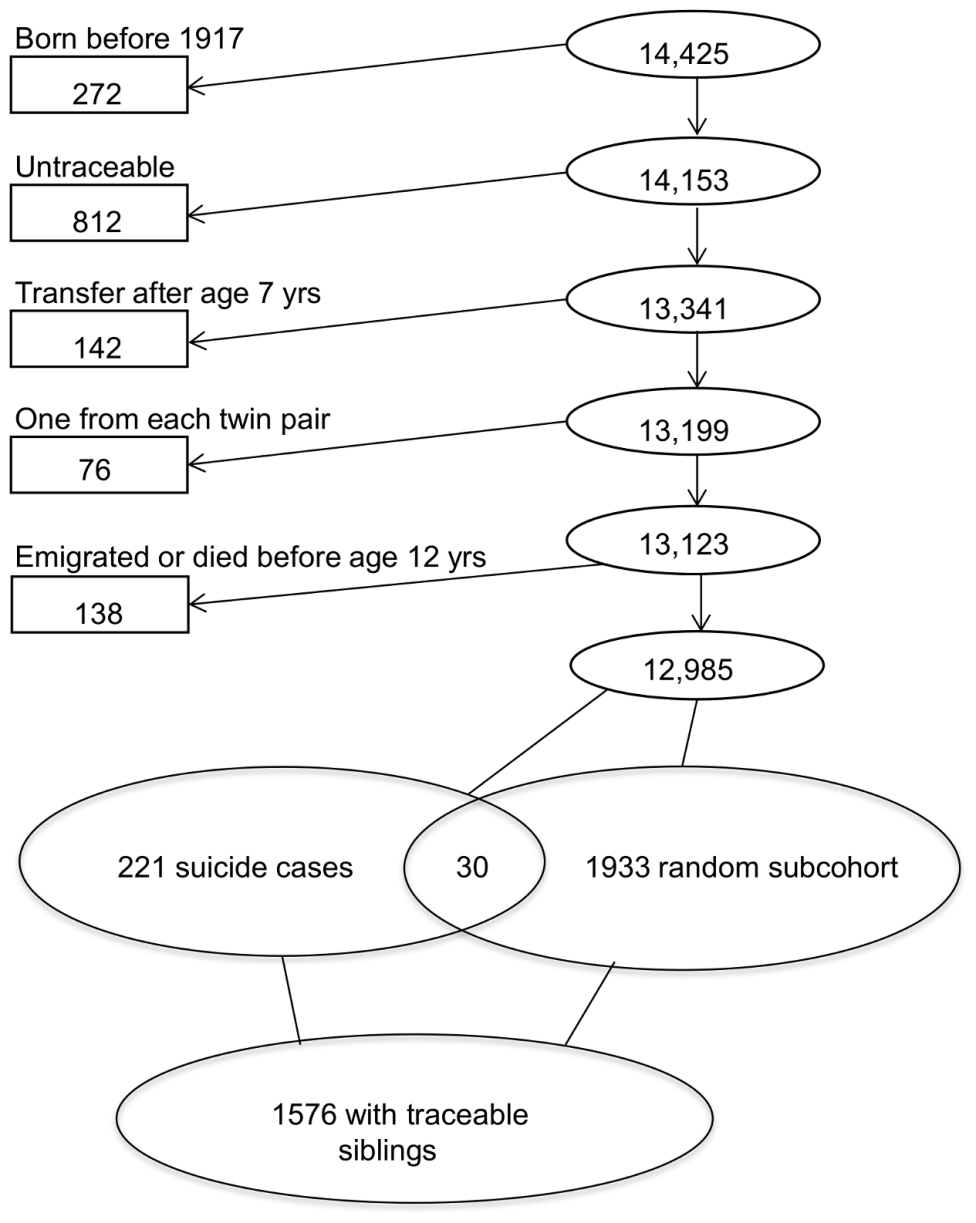
Flow-chart illustrating the formation of the study group by showing exclusions from the original cohort of adoptees.

As a robustness analysis and in order to increase power in the analyses, an alternative division into cohorts was applied; one cohort included siblings of adoptees dying by suicide before 31 December 2006, while the other cohort includes siblings of adoptees surviving until 31 December 2006, regardless of whether the suicides occurred before or after the age of 60 years. Allowing inclusion of suicides among adoptees occurring after the age of 60 years and before the end of follow-up by December 31, 2006, increased the number from 173 to 196 adoptees. By defining the comparison cohort based on adoptees who survived until the end of follow-up to December 31, 2006, reduced the size of this cohort from 1403 to 1110 adoptees due to death (and emigration) occurring after age 60 years. The flowchart in Figure 1 shows the formation of the study groups of 1576 adoptees, in which siblings were traced after various exclusions of the adoptees from the original cohort. The number of adoptees and siblings for the main and the robustness analyses, and follow-up periods are summarized in Table 1. There are some restrictions by design, as we analyzed siblings of individuals in a group of adoptees, where the adoptees were chosen by a case-cohort sample defined in 31 December 2006. The survival analysis targets survival times of the siblings, therefore covariates relating to the adoptee cannot be time dependent.

**Table 1:** Study design.

First cohort	Siblings of adoptees dying by suicide before age 60 years	Sibling of adoptees dying by suicide before 2006
Comparison cohort	Siblings of adoptees surviving to age 60 years	Sibling of adoptees surviving to 2006
Number of included adoptees with siblings	173 suicides 1403 survivors	196 suicides 1110 survivors
Follow up of adoptees	From 1 Jan 1917 until 1 April 2006	From 1 Jan 1917 until 1 April 2006
Number of included siblings	6,178 87 suicides	5,139 60 suicides
Follow up of siblings	From 1 Jan 1895 until 31 Dec 2009	From 1 Jan 1895 until 31 Dec 2009

### Outcome

The Danish regional population registers cover the period from 1924 through 1968 in paper files. From April 1968 a computerized national register exists, in which the parents and the children are linked [17]. From these sources, 6,178 siblings born before 1 April 1993 were identified and traced. The cause of death was known for those who died before 31 December 2009. These siblings were followed either until death or until censoring on 31 December 2009, whichever came first. In the analyses, the event was defined as death by suicide.

### Statistical methods

Cox regression models were used to estimate rate ratios (with 95% confidence intervals (CIs)) of suicide among siblings in the two cohorts. Using an indicator for being in one or the other cohort provided estimates of the suicide rate among siblings of adoptees dying by suicide before age 60 years compared to the suicide rate among siblings of adoptees surviving to age 60 years. The Cox models used age as time variable and were stratified by sex of the adoptee and of the siblings, and by three intervals of years of birth, defined to be of approximately equal size (for adoptees, 1917-36, 1937-43, 1944-47, and for siblings, 1895-1938, 1939-47, 1948-78), i.e. there are different underlying hazards in the stratification groups. In additional analyses, adoptees were excluded if they had a psychiatric admission at any time during the observation period (i.e. adjustment for psychiatric admissions in the adoptees was handled by restricting analyses to those without psychiatric admission). Among the siblings, psychiatric admissions were adjusted for by treating them as time-dependent co-variables (i.e. the risk time for a sibling was split into the time before any psychiatric admission and the time after the first psychiatric admission). The proportional hazards assumptions were tested by Schoenfeld´s residuals and were not rejected (p>0.20). All siblings, that is biologically full and paternal and maternal half siblings and adoptive siblings, were included in the same model with an indicator co-variable for their sibling status. Siblings of the same adoptee comprised a cluster, and allowance for possible within-cluster dependence was made by using robust standard errors. Analyses were carried out by the computer package Stata version 12.0 (StataCorp, College Station, TX). 

### Ethics statement

Since the study is entirely based on register data, there was according to Danish law no request for an ethical permission. The study was approved by Danish Data Protection Agency.

## Results

Table 2 shows the occurrence of suicide in biological and adoptive siblings of adoptees who died by suicide before the age of 60 years or survived to age 60 years. Table 3 presents associations between suicide in adoptees and rate of suicide in siblings. In full siblings a statistically significant excess rate of suicides was found among siblings of adoptees dying by suicide before age 60 years versus siblings of adoptees surviving to age 60 years. The excess rate in maternal half siblings was not statistically significant. In adoptive siblings the rate ratio could not be estimated as no one died by suicide among those having an adoptive sibling who died by suicide (confer the 0 cell in Table 2), suggesting no increase in risk of suicide given suicide in the adoptee. We found no statistically significant difference in effect on male and female biological siblings (p>0.12) and no statistically significant difference in effect on biological siblings under or above age 30 years (p>0.82).

**Table 2:** Occurrence of suicides in biological and adoptive siblings of adoptees who died by suicide before age 60 years or survived to age 60 years.

		Sibling of surviving adoptees	Siblings of adoptees dying by suicide
Full siblings	Surviving	676	113
	Suicide	5	4
	Rate per 1000 years	0.14	0.66
Paternal half siblings	Surviving	1928	225
	Suicide	22	3
	Rate per 1000 years	0.22	0.25
Maternal half siblings	Surviving	2108	185
	Suicide	30	6
	Rate per 1000 years	0.28	0.64
Adoptive siblings	Surviving	752	104
	Suicide	17	0
	Rate per 1000 years	0.42	0.00

Occurrence is represented by absolute numbers and as crude rates calculated as cases per person years.

**Table 3:** Rate ratio for suicide among biological and adoptive siblings, relative to suicide before age 60 years in the adoptees (reference group consists of siblings of adoptees who were known to be alive at the age of 60 years).

	Biological siblings		
	Full siblings	Paternal half siblings	Maternal half siblings
Adoptee suicide	5.01 (1.28 - 19.6)	1.03 (0.30 - 3.52)	2.25 (0.89 - 5.70)
Adoptee suicide, adjusted for psychiatric admission of the siblings	4.19 (1.00 - 17.5)	0.70 (0.16 - 3.00)	1.99 (0.75 - 5.31)
Adoptee suicide, adjusted for psychiatric admission of both siblings and adoptees^a^	13.9 (1.85 - 104)	-	2.60 (0.66 - 10.2)

Analyses are stratified on sex of adoptee and sibling, and by birth year groups of adoptee and sibling. Siblings of the same adoptee were included in one model allowing for correlation between observations. The rate ratios for adoptive siblings could not be estimated because there were no deaths by suicide among the exposed adoptive siblings.

^a^Restricted to siblings of adoptees who were not registered with a psychiatric admission

Adjustment for psychiatric illness of the siblings attenuated the associations, but they remained significant for biological full siblings. When the comparison was restricted to adoptees without a psychiatric admission, together with adjustment for psychiatric admission in the siblings, the elevated rate of suicide in full biological siblings was increased to 13.9, but with a very wide confidence interval. The non-significantly elevated rate of suicide in maternal half siblings remained. 

In the robustness analyses of siblings of adoptees dying by suicide before 2006 versus those surviving to 2006, the rate ratios found were 8.66 (1.53 - 48.9) in full siblings, 1.49 (0.47 - 4.74) in paternal half siblings, 2.73 (1.12 - 6.65) in maternal half siblings, and 0.93 (0.12 - 7.16) in adoptive siblings.

## Discussion

This adoption study of genetic and familial environmental influences on suicide has provided clear evidence of genetic transmission of suicide risk. Depending on the time period for observing suicides in the adoptees, this risk in full biological siblings of adoptees who had died by suicide was elevated five and eight times compared to the risk in biological full siblings of adoptees not dying by suicide. We found no evidence of environmental influence of adoptive families, since there was no correlation between adoptee outcome and risk in their adoptive siblings. These results corroborate the findings of earlier twin and adoptive family studies [3,9,10]. The increased risk for biological full siblings found in our study is higher than in the Swedish study of biological parents and adoptees [10]. This could be due to several factors, for example, non-additive genetic effects and confounding from generational changes in environmental factors when studying parents and children instead of siblings. The findings from our study and that of Tidemalm et al. differed in terms of the influence of the shared environment on suicide risk [6]. This could be due to lack of statistical power in our study, or it could be due to the fact that non-additive genetic influences and non-paternity biased the estimate of the influence of shared environment in the Swedish study. Though the study included as many adoptive siblings as biological full siblings, from the analyses where adoptive siblings were followed up until 2006, which was the only period where the risk in adoptive siblings of adoptees who had died by suicide compared to the risk in adoptive siblings of adoptees not dying by suicide could be estimated, the estimated rate ratio was 0.93 (0.12 - 7.16). It is evident from the wide confidence interval that our study lacked power to exclude there being an influence from shared environment. Our results confirmed that the elevated risk of suicide in biological siblings of adoptees is largely independent of a family history of psychiatric disorder leading to hospital admissions. 

In contrast to earlier studies we did not include parents, but instead focused on siblings, and divided them into full, paternal half and maternal half siblings. The results suggested that maternal transmission of suicide risk may be greater than paternal transmission, as also found in the general Nordic populations [6,18]. However, non-paternity might have influenced this finding by reducing the strength of the associations on the fathers’ side as compared to the mothers’ side. According to Kety and colleagues [19], the accuracy of identification of biological fathers of adoptees is probably greater than is usually the case since the law requires putative fathers to acknowledge paternity and to contribute to the costs of care of their children. Stronger associations between risks in adoptees and their maternal half-siblings than between adoptees and their paternal half-siblings could also come about by pre-adoptive (most likely prenatal) influences of the maternal environment. This may contribute to the associations between adoptees and full siblings, but the differences in associations with the full siblings and with the maternal half-siblings indicate that genetic contributions are more important than the maternal environment equally shared by the two types of siblings. 

Although the genetic associations appeared independent of psychiatric admissions, the mechanisms involved in genetic transmission of risk of suicide may imply undiagnosed psychiatric conditions or psychological pathways under genetic control. Likely candidates include transmission of personality traits associated with suicide such as aggression and impulsivity [20]. There is reasonable evidence that suicide risk is increased in the presence of reduced levels of 5-hydroxyindoleacetic acid [21-23], which is also known to be associated with aggression [22]. This may be a mediator of genetic transmission, given the known association of increased risk of suicide in individuals with certain polymorphisms of genes influencing the serotonergic transmission system [24].

### Strengths and limitations

This is a relatively large study involving a national consecutive sample of adoptees with a long follow-up period. Use of register data enables collection of follow-up information on all individuals in the cohort except the approximately one percent who emigrate. Restricting the study to siblings of adoptees reduced the possible confounding effects of temporal or cohort influences possibly affecting studies of adoptees and their parents. Psychiatric history was defined in terms of psychiatric admission, which is clearly a relatively crude measure of psychiatric disorder.

We excluded familial adoptions in order to allow clearer evaluation of any effects as indicative of genetic influence. To exclude familial adoptions, such as aunts or uncles of the adoptees, requires either that the information is gathered from adoption papers or, if the exclusion is based on register-based information, linkage to the grandparents of the adoptees is required. The proportions of individuals excluded as familial adoptions in the Swedish data were surprisingly low (about 3%) [10], which could indicate that the linkage available does not allow complete exclusion of familial adoptions. In the Danish data from 1924 to 1947, the familial adoptions which were excluded comprised approximately 50% of the total number of adoptions [25]. 

Generalizing results from twin and adoption studies to the general population requires caution, because risk of suicide appears to be reduced in twins compared to the general population [26], and elevated in adoptees [9,27]. However, the finding of genetic influence on suicides in both low and high risk populations allows more affirmative conclusions. In our sample of adoptive siblings of surviving adoptees the rate of suicide was high compared to that in biological siblings of surviving adoptees. This may be an indication of some selection bias in addition to lack of power, and makes the finding of no environmental influence on suicide risk questionable.

## Conclusions

Genetic influences make a major contribution to risk of suicide, which may occur independent of known psychiatric disorder. This is important because it not only increases our understanding of influences on risk of suicide, but also has implications for provision of advice to families with a history of suicide, including possible preventive initiatives. Also, future studies should, where possible, include control for all psychiatric disorders, not just those identified by history of psychiatric admission.
