# Correction: Genetic and Familial Environmental Effects on Suicide – An Adoption Study of Siblings

**DOI:** 10.1371/annotation/41113674-7ca2-42a5-a364-f646ff85c2e7

**Published:** 2014-01-06

**Authors:** Liselotte Petersen, Thorkild I. A. Sørensen, Per Kragh Andersen, Preben Bo Mortensen, Keith Hawton

Affiliations numbers 1 and 2 for the first, second and fourth authors are incorrect. The correct affiliation number 1 is: Institute of Preventive Medicine, Bispebjerg and Frederiksberg Hospital, Capital Region, Copenhagen, Denmark. The correct affiliation number 2 is: National Centre for Register-based Research, University of Aarhus, Aarhus, Denmark. 

